# Genome Sequence of an Indigoid-Producing Strain, *Pseudomonas* sp. PI1

**DOI:** 10.1128/genomeA.00622-15

**Published:** 2015-06-11

**Authors:** Yuanyuan Qu, Ziyan Liu, Wenli Shen, Shuzhen Li, Hongzhi Tang, Ping Xu

**Affiliations:** a Key Laboratory of Industrial Ecology and Environmental Engineering, School of Environmental Science and Technology, Dalian University of Technology, Dalian, People’s Republic of China; b State Key Laboratory of Microbial Metabolism and School of Life Sciences and Biotechnology, Shanghai Jiao Tong University, Shanghai, People’s Republic of China

## Abstract

*Pseudomonas* sp. strain PI1 can cometabolize indole in the presence of phenol to produce various indigoids. Here, we present a 7.2-Mb draft genome sequence of strain PI1, which may provide insight into the study of phenol-indole cometabolism and its application in aromatic bioremediation and wastewater treatment processes.

## GENOME ANNOUNCEMENT

Indigoids have been widely applied in the dye, food, cosmetic, and pharmaceutical industries. Compared with plant extraction and chemical synthesis, microbial production of indigoids has attracted much attention due to the merits of environmentally benign and mild reaction conditions (1, 2). Numerous wide microorganisms can convert indole into indigo with or without the coexistence of aromatic compounds (3–5). Among the various microbial resources, phenol-degrading strains showed unique characteristics (5). For example, *Acinetobacter* sp. ST-500 can produce relatively high indigo yields in diphenylmethane-water two-phase systems (6). Phenol hydroxylase from *Pseudomonas* sp. KL33 and *Pseudomonas* sp. KL28 catalyzed the production of various indigoid dyestuffs and hydroxyindoles from indole derivatives (7). Previously, we also cloned and expressed a novel phenol hydroxylase from *Arthrobacter* sp. W1 and revealed a novel indigoid formation pathway (8). Recently, a Gram-negative phenol-degrading bacterial strain named PI1 was isolated from lab bioreactors. It was identified as a member of the genus *Pseudomonas* according to 16S rRNA gene analysis. *Pseudomonas* sp. PI1 can cometabolize indole in the presence of phenol but cannot use indole as the sole carbon source (9). Application of liquid-chromatography time-of-flight mass spectrometry revealed four products with an *m/z* of 262.067, all of which had the same molecular formula with indigo, i.e., C_16_H_10_N_2_O_2_. Results also showed that the proportion of the products varied with different indole concentrations. All these results indicate that new pathways exist for indole cometabolism in strain PI1. The genome report of strain PI1 will provide genetic information for exploring phenol-indole cometabolism study.

The genome sequence of *Pseudomonas* sp. PI1 was obtained using an Illumina HiSeq-2000 sequencer (101 bp for each read). The reads were assembled *de novo* into 105 contigs using Velvet 1/2/10 software (10). The genome annotation was performed using the Rapid Annotations using Subsystems Technology (RAST) annotation server (11). The genome sequence of strain PI1 is 7,164,172 bp in length. A total of 6,288 candidate protein-coding sequences (CDSs) were predicted with coding intensity of 83.1%. There are 550 subsystems and 61 RNA genes present in the genome sequence.

The complete *dmpKLMNOP* components were identified in the genome, and *dmpN* showed 80% similarity with that of *Pseudomonas* sp. CF600 and *Pseudomonas* sp. KL33, suggesting that they might have similar indole transformation properties which needed further verification. In addition, a total of 225 CDSs were annotated for metabolism of aromatic compounds including biphenyl, salicylate, benzoate, *p*-hydroxybenzoate, and chloroaromatics. Interestingly, a rich set of annotated CDSs (107) were responsible for nitrogen metabolism, among which, 40 were for denitrification and 30 for ammonia assimilation, implying that strain PI1 might be a promising aerobic denitrifying bacterium that could be used in wastewater treatment processes. The genome information of strain PI1 reported here will provide sufficient information for the study of phenol-indole cometabolism and its application in aromatic bioremediation and wastewater treatment processes.

### Nucleotide sequence accession numbers.

This whole-genome shotgun project has been deposited at DDBJ/EMBL/GenBank under the accession no. JWMC00000000. The version described in this paper is the first version, JWMC00000000.1.
